# Supplementary figures and images for: CESCProg: a compact prognostic model and nomogram for cervical cancer based on miRNA biomarkers
Source: PeerJ. 2023 Sep 27;11:e15912. doi: 10.7717/peerj.15912 (PMC10541812; doi:10.7717/peerj.15912)

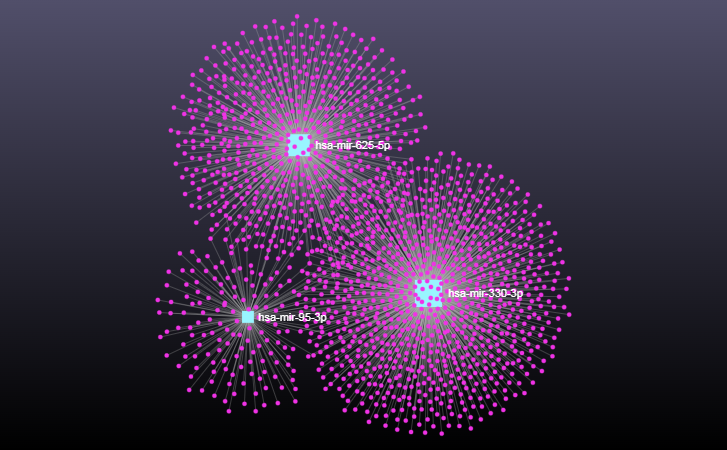

Supplement: Supplemental Information 3 [file peerj-11-15912-s003.zip › S3-NetworkEnrichment/Enrichment_results/miRnet.png]
